# The genome sequence of the Plumed Fan-foot, *Polypogon plumigeralis* (Hübner, 1825) (Lepidoptera: Erebidae)

**DOI:** 10.12688/wellcomeopenres.25062.1

**Published:** 2025-11-05

**Authors:** David C. Lees, Natural History Museum Genome Acquisition Lab, Darwin Tree of Life Barcoding Collective, Wellcome Sanger Institute Tree of Life Management, Samples and Laboratory team, Wellcome Sanger Institute Scientific Operations: Sequencing Operations, Wellcome Sanger Institute Tree of Life Core Informatics team, Tree of Life Core Informatics collective, Darwin Tree of Life Consortium

**Affiliations:** 1 Natural History Museum, London, England, UK

**Keywords:** Polypogon plumigeralis; Plumed Fan-foot; genome sequence; chromosomal; Lepidoptera

## Abstract

We present a genome assembly from an individual male *Polypogon plumigeralis* (Plumed Fan-foot; Arthropoda; Insecta; Lepidoptera; Erebidae). The assembly contains two haplotypes with total lengths of 639.54 megabases and 639.39 megabases. Most of the haplotype 1 sequence (99.54%) is scaffolded into 31 chromosomal pseudomolecules, including the Z sex chromosome. Haplotype 2 was assembled to scaffold level. The mitochondrial genome has also been assembled, with a length of 16.98 kilobases. This assembly was generated as part of the Darwin Tree of Life project, which produces reference genomes for eukaryotic species found in Britain and Ireland.

## Species taxonomy

Eukaryota; Opisthokonta; Metazoa; Eumetazoa; Bilateria; Protostomia; Ecdysozoa; Panarthropoda; Arthropoda; Mandibulata; Pancrustacea; Hexapoda; Insecta; Dicondylia; Pterygota; Neoptera; Endopterygota; Amphiesmenoptera; Lepidoptera; Glossata; Neolepidoptera; Heteroneura; Ditrysia; Obtectomera; Noctuoidea; Erebidae; Herminiinae; *Polypogon*; *Polypogon plumigeralis* (Hübner, 1825) (NCBI:txid997515)

## Background

*Polypogon plumigeralis* (Hübner, [1825]), also known as the Plumed Fanfoot ( Waring *et al.*, 2017), formerly placed in the genus *Pechipogo* Hübner, [1825] ( Yela *et al.*, 1997), is a moth in the family Erebidae (subfamily Herminiinae) with around 15–17 mm wingspan ( Waring *et al.*, 2017). This light brown moth has a slightly falcate apex; the forewing subterminal cross-line is fairly straight, not reaching the apex, inwardly darker and outwardly whitish. A faintly darker median fascia contains a darkish crescent (reniform stigma) and is outwardly bordered indistinctly with a line that is outwardly curved around this stigma; the innermost line is jagged. The light brown hindwing has two curved crosslines, the outer one bordered whitish. As with other fanfoots, the elegant protruding labial palps are held together and recurved upward and outward at the tip, and the antennae are strongly pectinate in the male. Although the resting posture is roughly triangular, the hindwings are often partially displayed when at rest. A similar, but slightly darker, species is the Dusky Fan-Foot *Zanclognatha lunalis* (Wocke, 1850), although in the United Kingdom, this is still a rare immigrant.

The adult moth flies from mid-June to late October (likely in two or more generations) ( Randle *et al.*, 2019: 257) and can be disturbed by day, otherwise attracted to light. The larva apparently feeds on decaying vegetal detritus. The eggs are whitish to pinkish with purplish blotches; the larva is yellowish to brownish with pale dorsal spots on each segment and very cryptic, and the pupa is reddish brown ( Lepiforum, 2025).

Up to the end of the last century, the species was a rare migrant to the south-east coast of the United Kingdom, being suspected of temporary colonisation ( Randle *et al.*, 2019). In recent years the moth has been recorded in greater numbers and more widely, notably since about 2018 when it became resident in south-west London, and currently there are 868 records for the UK on the NBN Gateway ( NBN Atlas Partnership, 2025). In Europe, most records ( GBIF Secretariat, 2025) are for western Europe, the range extending southeast to Cyprus with a couple of records north and south of the Black Sea, and although currently with 625 records, this map is not yet updated for the recent expansion in the UK.

The DNA barcode extracted from the mitogenome assembly (OZ210444.1) is identical to sequences from European specimens on BOLD (accessed 30 June 2025), specifically from Austria and Italy, and falls within the COI-5P cluster BOLD:AAI4196. A specimen from Cyprus (BC_ZSM_Lep_104746), assigned to a separate cluster (BOLD:ADA7410), differs by 3.65% *p*-distance. Although morphologically similar, this Cypriot specimen may not be conspecific. Notably, the North American Morbid Owlet Moth *Chytolita morbidalis* (Guenée, 1854), currently regarded as monotypic, also shows similar levels of divergence from BOLD:ADA7410. By contrast, the DNA barcode of *Phalaena strigilata* L. (Common Fanfoot), the type species of *Pechipogo*, is approximately 4.85% divergent from OZ210444.1. *Polypogon plumigeralis* appears not to have been included in previous molecular studies, and the genome presented here may assist in identifying its sister taxon. It may also provide evidence relevant to reassessing its current generic placement – which has been debated ( Fibiger *et al.*, 2010) – within *Polypogon* Schrank, 1802, whose type species is *Phalaena* ( *Geometra*) *tentacularia* L., 1758.

**Figure 1.**
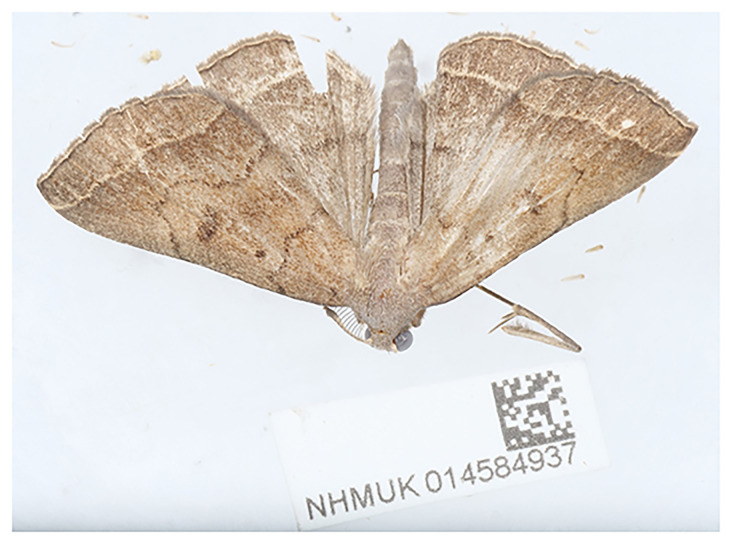
Photograph of the *Polypogon plumigeralis* (ilPolPlum1) specimen used for genome sequencing.

The assembly presented here was produced using the Tree of Life pipeline from a specimen collected in Natural History Museum, London, England ( Figure 1), as part of the Darwin Tree of Life project.

## Methods

### Sample acquisition and DNA barcoding

The specimen used for genome sequencing was an adult male *Polypogon plumigeralis* (specimen ID NHMUK014584937, ToLID ilPolPlum1; Figure 1), collected from Natural History Museum, London, England, United Kingdom (latitude 51.5, longitude –0.18) on 2022-09-15. The specimen was collected and identified by David Lees (Natural History Museum London). Sample metadata were collected in line with the Darwin Tree of Life project standards described by Lawniczak *et al.* (2022).

The initial identification was verified by an additional DNA barcoding process according to the framework developed by Twyford *et al.* (2024). A small sample was dissected from the specimen and stored in ethanol, while the remaining parts were shipped on dry ice to the Wellcome Sanger Institute (WSI) (see the protocol). The tissue was lysed, the COI marker region was amplified by PCR, and amplicons were sequenced and compared to the BOLD database, confirming the species identification ( Crowley *et al.*, 2023). Following whole genome sequence generation, the relevant DNA barcode region was also used alongside the initial barcoding data for sample tracking at the WSI ( Twyford *et al.*, 2024). The standard operating procedures for Darwin Tree of Life barcoding are available on protocols.io.

### Nucleic acid extraction

Protocols for high molecular weight (HMW) DNA extraction developed at the Wellcome Sanger Institute (WSI) Tree of Life Core Laboratory are available on protocols.io ( Howard *et al.*, 2025). The ilPolPlum1 sample was weighed and triaged to determine the appropriate extraction protocol. Tissue from the thorax was homogenised by powermashing using a PowerMasher II tissue disruptor. HMW DNA was extracted in the WSI Scientific Operations core using the Automated MagAttract v2 protocol. DNA was sheared into an average fragment size of 12–20 kb following the Megaruptor®3 for LI PacBio protocol. Sheared DNA was purified by automated SPRI (solid-phase reversible immobilisation). The concentration of the sheared and purified DNA was assessed using a Nanodrop spectrophotometer and Qubit Fluorometer using the Qubit dsDNA High Sensitivity Assay kit. Fragment size distribution was evaluated by running the sample on the FemtoPulse system. For this sample, the final post-shearing DNA had a Qubit concentration of 11.03 ng/μL and a yield of 518.41 ng, with a fragment size of 16.2 kb. The 260/280 spectrophotometric ratio was 1.73, and the 260/230 ratio was 2.31.

### PacBio HiFi library preparation and sequencing

Library preparation and sequencing were performed at the WSI Scientific Operations core. Libraries were prepared using the SMRTbell Prep Kit 3.0 (Pacific Biosciences, California, USA), following the manufacturer’s instructions. The kit includes reagents for end repair/A-tailing, adapter ligation, post-ligation SMRTbell bead clean-up, and nuclease treatment. Size selection and clean-up were performed using diluted AMPure PB beads (Pacific Biosciences). DNA concentration was quantified using a Qubit Fluorometer v4.0 (ThermoFisher Scientific) and the Qubit 1X dsDNA HS assay kit. Final library fragment size was assessed with the Agilent Femto Pulse Automated Pulsed Field CE Instrument (Agilent Technologies) using the gDNA 55 kb BAC analysis kit.

The sample was sequenced on a Revio instrument (Pacific Biosciences). The prepared library was normalised to 2 nM, and 15 μL was used for making complexes. Primers were annealed and polymerases bound to generate circularised complexes, following the manufacturer’s instructions. Complexes were purified using 1.2X SMRTbell beads, then diluted to the Revio loading concentration (200–300 pM) and spiked with a Revio sequencing internal control. The sample was sequenced on a Revio 25M SMRT cell. The SMRT Link software (Pacific Biosciences), a web-based workflow manager, was used to configure and monitor the run and to carry out primary and secondary data analysis.

### Hi-C

***Sample preparation and crosslinking***

The Hi-C sample was prepared from 20–50 mg of frozen thorax tissue of the ilPolPlum1 sample using the Arima-HiC v2 kit (Arima Genomics). Following the manufacturer’s instructions, tissue was fixed and DNA crosslinked using TC buffer to a final formaldehyde concentration of 2%. The tissue was homogenised using the Diagnocine Power Masher-II. Crosslinked DNA was digested with a restriction enzyme master mix, biotinylated, and ligated. Clean-up was performed with SPRISelect beads before library preparation. DNA concentration was measured with the Qubit Fluorometer (Thermo Fisher Scientific) and Qubit HS Assay Kit. The biotinylation percentage was estimated using the Arima-HiC v2 QC beads.

***Hi-C library preparation and sequencing***

Biotinylated DNA constructs were fragmented using a Covaris E220 sonicator and size selected to 400–600 bp using SPRISelect beads. DNA was enriched with Arima-HiC v2 kit Enrichment beads. End repair, A-tailing, and adapter ligation were carried out with the NEBNext Ultra II DNA Library Prep Kit (New England Biolabs), following a modified protocol where library preparation occurs while DNA remains bound to the Enrichment beads. Library amplification was performed using KAPA HiFi HotStart mix and a custom Unique Dual Index (UDI) barcode set (Integrated DNA Technologies). Depending on sample concentration and biotinylation percentage determined at the crosslinking stage, libraries were amplified with 10–16 PCR cycles. Post-PCR clean-up was performed with SPRISelect beads. Libraries were quantified using the AccuClear Ultra High Sensitivity dsDNA Standards Assay Kit (Biotium) and a FLUOstar Omega plate reader (BMG Labtech).

Prior to sequencing, libraries were normalised to 10 ng/μL. Normalised libraries were quantified again and equimolar and/or weighted 2.8 nM pools were created. Pool concentrations were checked using the Agilent 4200 TapeStation (Agilent) with High Sensitivity D500 reagents before sequencing. Sequencing was performed using paired-end 150 bp reads on the Illumina NovaSeq X.

### Genome assembly

Prior to assembly of the PacBio HiFi reads, a database of *k*-mer counts ( *k* = 31) was generated from the filtered reads using FastK. GenomeScope2 ( Ranallo-Benavidez *et al.*, 2020) was used to analyse the *k*-mer frequency distributions, providing estimates of genome size, heterozygosity, and repeat content.

The HiFi reads were assembled using Hifiasm in Hi-C phasing mode ( Cheng *et al.*, 2021; Cheng *et al.*, 2022), producing two haplotypes. Hi-C reads ( Rao *et al.*, 2014) were mapped to the primary contigs using bwa-mem2 ( Vasimuddin *et al.*, 2019). Contigs were further scaffolded with Hi-C data in YaHS ( Zhou *et al.*, 2023), using the --break option for handling potential misassemblies. The scaffolded assemblies were evaluated using Gfastats ( Formenti *et al.*, 2022), BUSCO ( Manni *et al.*, 2021) and MERQURY.FK ( Rhie *et al.*, 2020).

The mitochondrial genome was assembled using MitoHiFi ( Uliano-Silva *et al.*, 2023), which runs MitoFinder ( Allio *et al.*, 2020) and uses these annotations to select the final mitochondrial contig and to ensure the general quality of the sequence.

### Assembly curation

The assembly was decontaminated using the Assembly Screen for Cobionts and Contaminants ( ASCC) pipeline. TreeVal was used to generate the flat files and maps for use in curation. Manual curation was conducted primarily in PretextView and HiGlass ( Kerpedjiev *et al.*, 2018). Scaffolds were visually inspected and corrected as described by Howe *et al.* (2021). Manual corrections included 14 breaks and 48 joins. The curation process is documented at https://gitlab.com/wtsi-grit/rapid-curation. PretextSnapshot was used to generate a Hi-C contact map of the final assembly.

### Assembly quality assessment

The Merqury.FK tool ( Rhie *et al.*, 2020) was run in a Singularity container ( Kurtzer *et al.*, 2017) to evaluate *k*-mer completeness and assembly quality for both haplotypes using the *k*-mer databases ( *k* = 31) computed prior to genome assembly. The analysis outputs included assembly QV scores and completeness statistics.

The genome was analysed using the BlobToolKit pipeline, a Nextflow implementation of the earlier Snakemake version ( Challis *et al.*, 2020). The pipeline aligns PacBio reads using minimap2 ( Li, 2018) and SAMtools ( Danecek *et al.*, 2021) to generate coverage tracks. It runs BUSCO ( Manni *et al.*, 2021) using lineages identified from the NCBI Taxonomy ( Schoch *et al.*, 2020). For the three domain-level lineages, BUSCO genes are aligned to the UniProt Reference Proteomes database ( Bateman *et al.*, 2023) using DIAMOND blastp ( Buchfink *et al.*, 2021). The genome is divided into chunks based on the density of BUSCO genes from the closest taxonomic lineage, and each chunk is aligned to the UniProt Reference Proteomes database with DIAMOND blastx. Sequences without hits are chunked using seqtk and aligned to the NT database with blastn ( Altschul *et al.*, 1990). The BlobToolKit suite consolidates all outputs into a blobdir for visualisation. The BlobToolKit pipeline was developed using nf-core tooling ( Ewels *et al.*, 2020) and MultiQC ( Ewels *et al.*, 2016), with containerisation through Docker ( Merkel, 2014) and Singularity ( Kurtzer *et al.*, 2017).

## Genome sequence report

### Sequence data

**Figure 2.**
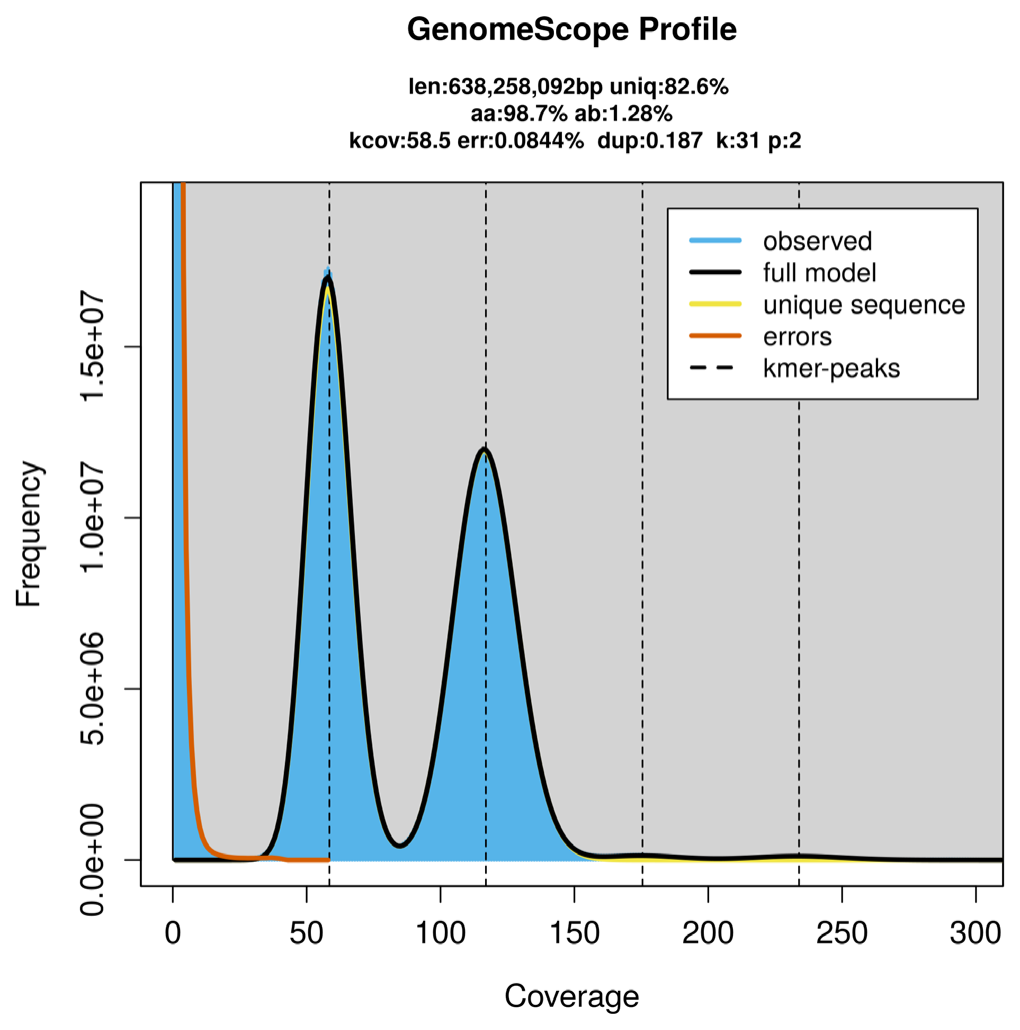
Frequency distribution of *k*-mers generated using GenomeScope2. The plot shows observed and modelled *k*-mer spectra, providing estimates of genome size, heterozygosity, and repeat content based on unassembled sequencing reads.

PacBio sequencing of the *Polypogon plumigeralis* specimen generated 76.88 Gb (gigabases) from 8.44 million reads, which were used to assemble the genome. GenomeScope2.0 analysis estimated the haploid genome size at 638.26 Mb, with a heterozygosity of 1.28% and repeat content of 17.39% ( Figure 2). These estimates guided expectations for the assembly. Based on the estimated genome size, the sequencing data provided approximately 117× coverage. Hi-C sequencing produced 157.65 Gb from 1 044.03 million reads, which were used to scaffold the assembly. Table 1 summarises the specimen and sequencing details.

**Table 1.** Specimen and sequencing data for BioProject PRJEB81654.

Platform	PacBio HiFi	Hi-C
**ToLID**	ilPolPlum1	ilPolPlum1
**Specimen ID**	NHMUK014584937	NHMUK014584937
**BioSample (source individual)**	SAMEA115574949	SAMEA115574949
**BioSample (tissue)**	SAMEA115575095	SAMEA115575095
**Tissue**	thorax	thorax
**Instrument**	Revio	Illumina NovaSeq X
**Run accessions**	ERR13900476	ERR13907249; ERR13907250
**Read count total**	8.44 million	1 044.03 million
**Base count total**	76.88 Gb	157.65 Gb

### Assembly statistics

The genome was assembled into two haplotypes using Hi-C phasing. Haplotype 1 was curated to chromosome level, while haplotype 2 was assembled to scaffold level. The final assembly has a total length of 639.54 Mb in 125 scaffolds, with 128 gaps, and a scaffold N50 of 21.36 Mb ( Table 2).

**Table 2.** Genome assembly statistics.

**Assembly name**	ilPolPlum1.hap1.1	ilPolPlum1.hap2.1
**Assembly accession**	GCA_964656525.1	GCA_964656545.1
**Assembly level**	chromosome	scaffold
**Span (Mb)**	639.54	639.39
**Number of** **chromosomes**	31	Scaffold-level
**Number of contigs**	253	204
**Contig N50**	9.18 Mb	8.03 Mb
**Number of scaffolds**	125	109
**Scaffold N50**	21.36 Mb	21.42 Mb
**Longest scaffold** **length (Mb)**	30.22	-
**Sex chromosomes**	Z	-
**Organelles**	Mitochondrion: 16.98 kb	-

**Figure 3.**
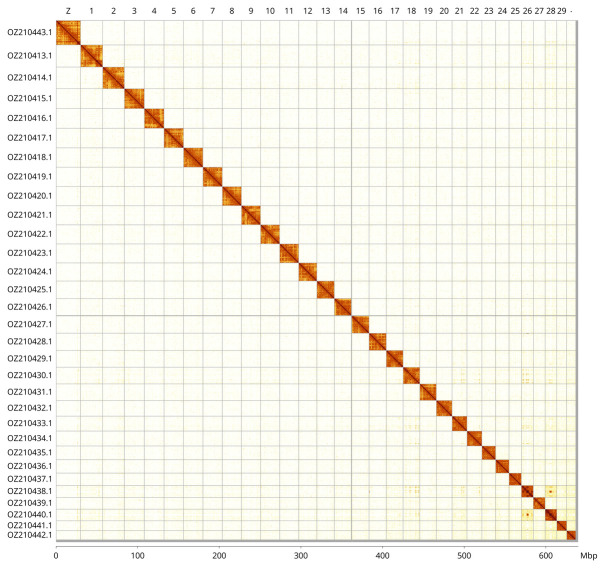
Hi-C contact map of the *Polypogon plumigeralis* genome assembly. Assembled chromosomes are shown in order of size and labelled along the axes, with a megabase scale shown below. The plot was generated using PretextSnapshot.

Most of the assembly sequence (99.54%) was assigned to 31 chromosomal-level scaffolds, representing 30 autosomes and the Z sex chromosome. These chromosome-level scaffolds, confirmed by Hi-C data, are named according to size ( Figure 3; Table 3). Chromosome Z was identified based BUSCO gene painting with ancestral Merian elements ( https://doi.org/10.1038/s41559-024-02329-4).”..

**Table 3.** Chromosomal pseudomolecules in the haplotype 1 genome assembly of *Polypogon plumigeralis* ilPolPlum1.

INSDC accession	Molecule	Length (Mb)	GC%
OZ210413.1	1	27.08	36
OZ210414.1	2	26.45	35.50
OZ210415.1	3	24.52	35.50
OZ210416.1	4	24.02	35.50
OZ210417.1	5	24.01	35.50
OZ210418.1	6	23.73	35.50
OZ210419.1	7	23.70	35.50
OZ210420.1	8	23.49	35.50
OZ210421.1	9	23.43	35.50
OZ210422.1	10	23.33	35.50
OZ210423.1	11	23.23	35.50
OZ210424.1	12	22.34	35.50
OZ210425.1	13	21.36	35.50
OZ210426.1	14	21.35	36
OZ210427.1	15	21.27	35.50
OZ210428.1	16	21.12	35.50
OZ210429.1	17	20.54	36
OZ210430.1	18	20.44	36
OZ210431.1	19	20.26	35.50
OZ210432.1	20	19.19	36
OZ210433.1	21	18.33	36
OZ210434.1	22	18.32	35.50
OZ210435.1	23	16.73	35.50
OZ210436.1	24	16.46	36
OZ210437.1	25	15.10	36
OZ210438.1	26	14.66	38
OZ210439.1	27	14.35	36
OZ210440.1	28	14.31	39
OZ210441.1	29	12.17	36.50
OZ210442.1	30	11.07	36.50
OZ210443.1	Z	30.22	35.50

The mitochondrial genome was also assembled. This sequence is included as a contig in the multifasta file of the genome submission and as a standalone record.

**Figure 4.**
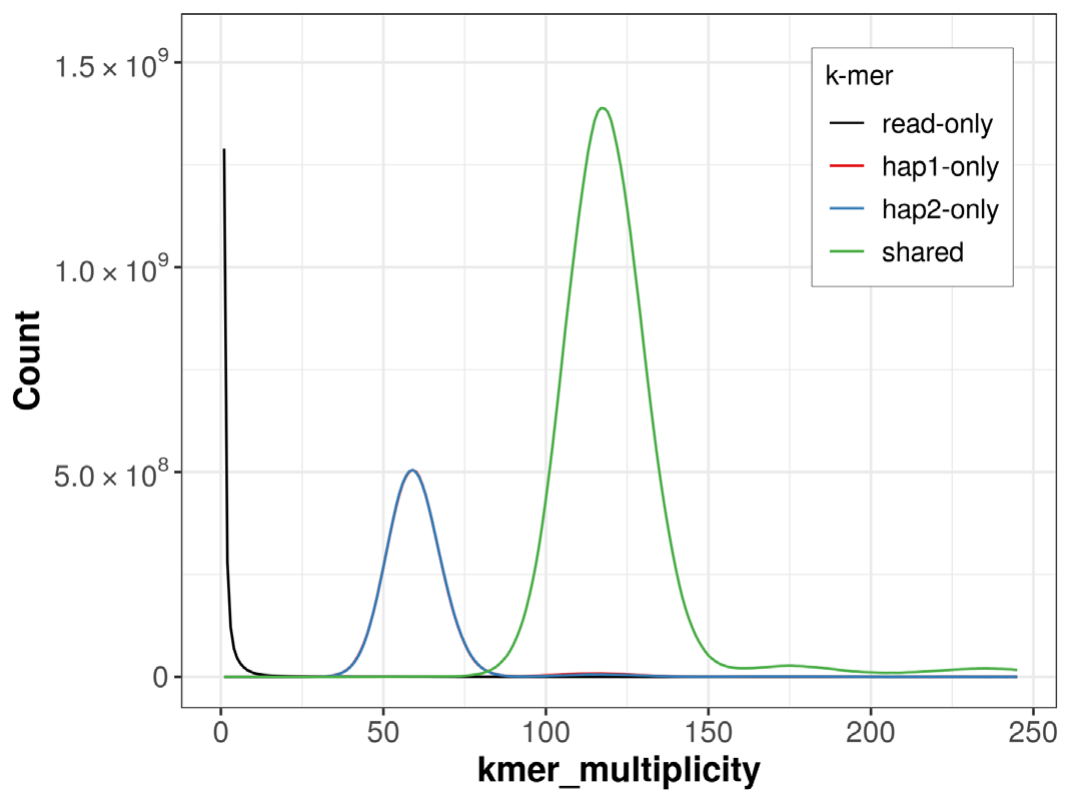
Evaluation of *k*-mer completeness using MerquryFK. This plot illustrates the recovery of *k*-mers from the original read data in the final assemblies. The horizontal axis represents *k*-mer multiplicity, and the vertical axis shows the number of *k*-mers. The black curve represents *k*-mers that appear in the reads but are not assembled. The green curve corresponds to *k*-mers shared by both haplotypes, and the red and blue curves show *k*-mers found only in one of the haplotypes.

For haplotype 1, the estimated QV is 65.0, and for haplotype 2, 64.6. When the two haplotypes are combined, the assembly achieves an estimated QV of 64.8. The *k*-mer completeness is 75.45% for haplotype 1, 75.37% for haplotype 2, and 99.88% for the combined haplotypes ( Figure 4).

**Figure 5.**
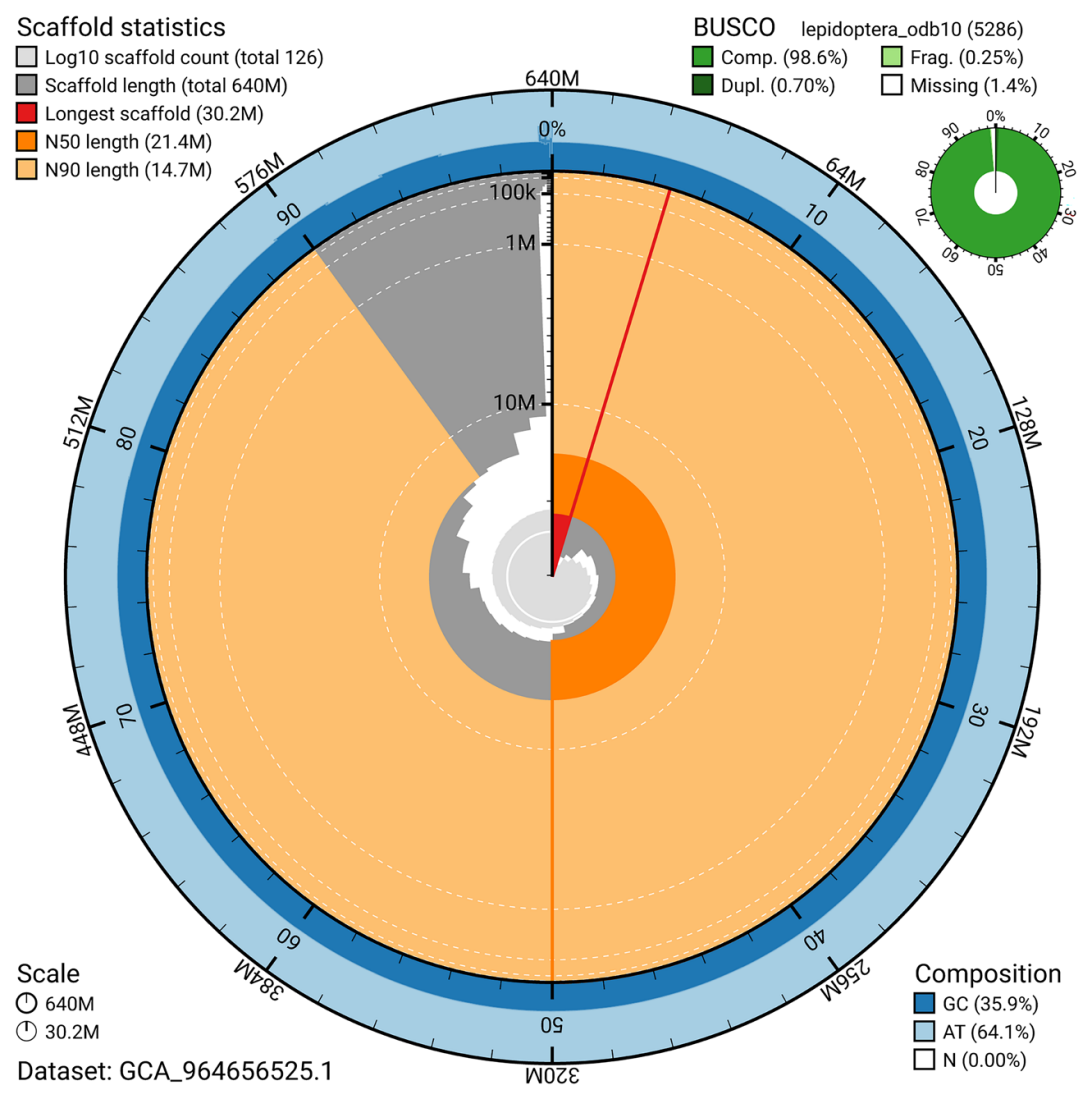
Assembly metrics for ilPolPlum1.hap1.1. The BlobToolKit snail plot provides an overview of assembly metrics and BUSCO gene completeness. The circumference represents the length of the whole genome sequence, and the main plot is divided into 1 000 bins around the circumference. The outermost blue tracks display the distribution of GC, AT, and N percentages across the bins. Scaffolds are arranged clockwise from longest to shortest and are depicted in dark grey. The longest scaffold is indicated by the red arc, and the deeper orange and pale orange arcs represent the N50 and N90 lengths. A light grey spiral at the centre shows the cumulative scaffold count on a logarithmic scale. A summary of complete, fragmented, duplicated, and missing BUSCO genes in the set is presented at the top right. An interactive version of this figure can be accessed on the BlobToolKit viewer.

**Figure 6.**
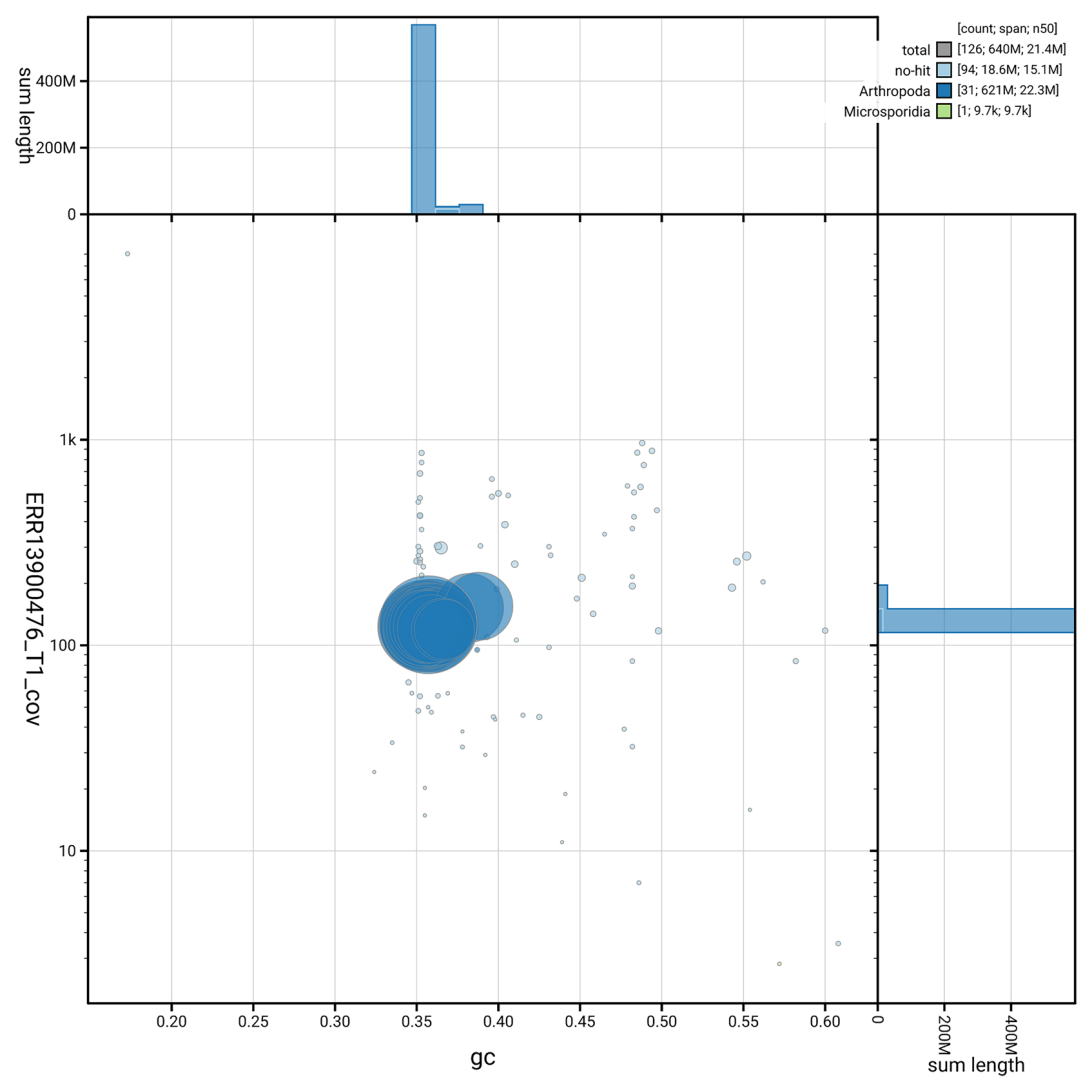
BlobToolKit GC-coverage plot for ilPolPlum1.hap1.1. Blob plot showing sequence coverage (vertical axis) and GC content (horizontal axis). The circles represent scaffolds, with the size proportional to scaffold length and the colour representing phylum membership. The histograms along the axes display the total length of sequences distributed across different levels of coverage and GC content. An interactive version of this figure is available on the BlobToolKit viewer.

BUSCO analysis using the lepidoptera_odb10 reference set ( *n* = 5 286) identified 98.6% of the expected gene set (single = 97.9%, duplicated = 0.7%) for haplotype 1. The snail plot in Figure 5 summarises the scaffold length distribution and other assembly statistics for haplotype 1. The blob plot in Figure 6 shows the distribution of scaffolds by GC proportion and coverage for haplotype 1.

Table 4 lists the assembly metric benchmarks adapted from Rhie *et al.* (2021) and the Earth BioGenome Project Report on Assembly Standards September 2024. The EBP metric, calculated for the haplotype 1, is **6.C.Q65**, meeting the recommended reference standard.

**Table 4.** Earth Biogenome Project summary metrics for the *Polypogon plumigeralis* assembly.

Measure	Value	Benchmark
EBP summary (haplotype 1)	6.C.Q65	6.C.Q40
Contig N50 length	9.18 Mb	≥ 1 Mb
Scaffold N50 length	21.36 Mb	= chromosome N50
Consensus quality (QV)	Haplotype 1: 65.0; haplotype 2: 64.6; combined: 64.8	≥ 40
*k*-mer completeness	Haplotype 1: 75.45%; Haplotype 2: 75.37%; combined: 99.88%	≥ 95%
BUSCO	C:98.6% [S:97.9%; D:0.7%]; F:0.2%; M:1.2%; n:5 286	S > 90%; D < 5%
Percentage of assembly assigned to chromosomes	99.54%	≥ 90%

### Wellcome Sanger Institute – Legal and Governance

The materials that have contributed to this genome note have been supplied by a Darwin Tree of Life Partner. The submission of materials by a Darwin Tree of Life Partner is subject to the **‘Darwin Tree of Life Project Sampling Code of Practice’**, which can be found in full on the Darwin Tree of Life website. By agreeing with and signing up to the Sampling Code of Practice, the Darwin Tree of Life Partner agrees they will meet the legal and ethical requirements and standards set out within this document in respect of all samples acquired for, and supplied to, the Darwin Tree of Life Project. Further, the Wellcome Sanger Institute employs a process whereby due diligence is carried out proportionate to the nature of the materials themselves, and the circumstances under which they have been/are to be collected and provided for use. The purpose of this is to address and mitigate any potential legal and/or ethical implications of receipt and use of the materials as part of the research project, and to ensure that in doing so we align with best practice wherever possible. The overarching areas of consideration are:

- Ethical review of provenance and sourcing of the material
- Legality of collection, transfer and use (national and international)

Each transfer of samples is further undertaken according to a Research Collaboration Agreement or Material Transfer Agreement entered into by the Darwin Tree of Life Partner, Genome Research Limited (operating as the Wellcome Sanger Institute), and in some circumstances, other Darwin Tree of Life collaborators.

## Data Availability

European Nucleotide Archive: Polypogon plumigeralis. Accession number PRJEB81654. The genome sequence is released openly for reuse. The *Polypogon plumigeralis* genome sequencing initiative is part of the Darwin Tree of Life Project (PRJEB40665), the Sanger Institute Tree of Life Programme (PRJEB43745) and Project Psyche (PRJEB71705). All raw sequence data and the assembly have been deposited in INSDC databases. The genome will be annotated using available RNA-Seq data and presented through the Ensembl pipeline at the European Bioinformatics Institute. Raw data and assembly accession identifiers are reported in Table 1 and Table 2. Production code used in genome assembly at the WSI Tree of Life is available at https://github.com/sanger-tol. Table 5 lists software versions used in this study.

## Author information

Contributors are listed at the following links:

- Members of the Natural History Museum Genome Acquisition Lab
- Members of the Darwin Tree of Life Barcoding collective
- Members of the Wellcome Sanger Institute Tree of Life Management, Samples and Laboratory team
- Members of Wellcome Sanger Institute Scientific Operations – Sequencing Operations
- Members of the Wellcome Sanger Institute Tree of Life Core Informatics team
- Members of the Tree of Life Core Informatics collective
- Members of the Darwin Tree of Life Consortium
