# Supplementary material for: The genome sequence of the Plumed Fan-foot, Polypogon plumigeralis (Hübner, 1825) (Lepidoptera: Erebidae)
Source: Wellcome Open Res. 2025 Nov 5;10:619. [Version 1] doi: 10.12688/wellcomeopenres.25062.1 (PMC12743215; doi:10.12688/wellcomeopenres.25062.1)
Supplement: Peer review history

# Reviewer report: Reviewer response for version 1

**DOI:** 10.21956/wellcomeopenres.27628.r140632

**Published:** 2025-12-26

**Authors:** Josephine R Paris

**Affiliations:** 1 University of Exeter Library, Devon, UK

This Data Note describes the genome assembly of the Plummed Fan-foot. The text is well written, with ample information on the Background of the species' biology and ecology. The Methods and Materials are well described and the datasets are clearly presented with relevant accession numbers. I have no specific comments.

Are sufficient details of methods and materials provided to allow replication by others?

Yes

Is the rationale for creating the dataset(s) clearly described?

Yes

Are the datasets clearly presented in a useable and accessible format?

Yes

Are the protocols appropriate and is the work technically sound?

Yes

Reviewer Expertise:

population genomics of non-models

I confirm that I have read this submission and believe that I have an appropriate level of expertise to confirm that it is of an acceptable scientific standard.

# Reviewer report: Reviewer response for version 1

**DOI:** 10.21956/wellcomeopenres.27628.r139781

**Published:** 2025-12-11

**Authors:** Panagiotis Ioannidis

**Affiliations:** 1 Foundation for Research & Technology - Hellas, Crete, Greece

This paper describes the sequencing and assembly of the genome of Polypogon plumigeralis, a lepidopteran insect.

 The introduction section is very detailed and complete, containing a lot of information about the ecology and biology of this species.

 The assembly of its genome is based on the familiar DToL pipeline and since it uses long reads and Hi-C sequencing data, it resulted in a very good assembly in terms of Merqury and BUSCO scores.

 The contig-to-chromosome (CC) ratio could be better; it currently is 122/31 = 3.93 and I've seen assemblies with lower CC ratio. The increased CC ratio is also showing in the blobplot where there are small contigs/scaffolds spread across non-expected GC ratios/coverage.

 Lastly, I read that the genome will be annotated "...using available RNA-seq data...". In my experience using RNAseq data from different species doesn't produce a good gene set. As a result I would urge the authors to make sure they evaluate the gene set they will get from such a pipeline using BUSCO and compare the results to those of the genome assembly. Such a comparison will make sure that no large numbers of conserved genes are missed.

 In summary it is a very good genome assembly, suitable for downstream analyses. Of course, not having a gene set limits the number of users that can actually take advantage of it, but hopefully a quality gene set will soon be available.

Are sufficient details of methods and materials provided to allow replication by others?

Yes

Is the rationale for creating the dataset(s) clearly described?

Yes

Are the datasets clearly presented in a useable and accessible format?

Yes

Are the protocols appropriate and is the work technically sound?

Yes

Reviewer Expertise:

insect genomics; bioinformatics

I confirm that I have read this submission and believe that I have an appropriate level of expertise to confirm that it is of an acceptable scientific standard.
